# An IMU Evaluation Method Using a Signal Grafting Scheme

**DOI:** 10.3390/s16060854

**Published:** 2016-06-10

**Authors:** Xiaoji Niu, Qiang Wang, You Li, Quan Zhang, Peng Jiang

**Affiliations:** 1 GNSS Research Center, Wuhan University, No.129, Luoyu Road, Wuhan 430079, China; xjniu@whu.edu.cn (X.N.); wang_qiang@whu.edu.cn (Q.W.); liyou331@gmail.com (Y.L.); zhangquan@whu.edu.cn (Q.Z.); 2 Department of Geomatics Engineering, University of Calgary, Calgary, AB T2N1N4, Canada; 3 Collaborative Innovation Center for Geospatial Information Technology, Wuhan University, No.129, Luoyu Road, Wuhan 430079, China

**Keywords:** signal grafting, performance evaluation, Inertial Measurement Units, hybrid simulation, navigation, field testing

## Abstract

As various inertial measurement units (IMUs) from different manufacturers appear every year, it is not affordable to evaluate every IMU through tests. Therefore, this paper presents an IMU evaluation method by grafting data from the tested IMU to the reference data from a higher-grade IMU. The signal grafting (SG) method has several benefits: (a) only one set of field tests with a higher-grade IMU is needed, and can be used to evaluate numerous IMUs. Thus, SG is effective and economic because all data from the tested IMU is collected in the lab; (b) it is a general approach to compare navigation performances of various IMUs by using the same reference data; and, finally, (c) through SG, one can first evaluate an IMU in the lab, and then decide whether to further test it. Moreover, this paper verified the validity of SG to both medium- and low-grade IMUs, and presents and compared two SG strategies, *i.e.*, the basic-error strategy and the full-error strategy. SG provided results similar to field tests, with a difference of under 5% and 19.4%–26.7% for tested tactical-grade and MEMS IMUs. Meanwhile, it was found that dynamic IMU errors were essential to guarantee the effect of the SG method.

## 1. Introduction

The complementary features of Global Navigation Satellite Systems (GNSS) and Inertial Navigation System (INS) have been investigated and exploited during the past decades [1]. GNSS can provide positioning solutions with long-term stability using the line-of-sight signals from GNSS satellites to the receiver. However, GNSS suffers from interruptions and degradations caused by various kinds of disturbances on the satellite signals [2,3]. On the contrary, INS is a self-contained system that ensures consistent availability of navigation information from an initial status, but has increasing errors in the long term, although in-field calibration can improve the navigation performance [4,5]. Therefore, GNSS and INS are often combined (especially when using low-cost systems) as an integrated navigation system. Due to the complementary characteristics of GNSS and INS, such an integrated system requires less accurate INS for general navigation applications and, thus, can minimize the limitations due to price, availability, and access restrictions of high-grade (e.g., navigation-grade) inertial measurement units (IMUs). Furthermore, advances in micro-electro-mechanical systems (MEMS) technology combined with the miniaturization of electronics have made it possible to produce chip-based inertial sensors for measuring angular rates and accelerations. These MEMS chips are small and lightweight, consume very little power, and are extremely low-cost. Therefore, several applications, such as motion tracking, outdoor/indoor navigation, and virtual reality, have been directed towards using MEMS sensors. Currently, various MEMS inertial sensors and IMUs developed by different manufacturers have emerged to market. For navigation applications of these sensors or IMUs, evaluating the corresponding navigation performance is important [6,7,8,9].

Common methods for IMU evaluation include lab calibration, INS simulation, and field testing [10,11]. These methods are developed for different purposes. Specifically, lab calibration is an efficient way to obtain the characteristics of IMUs; INS simulation is suitable for evaluating the impact of one single error source; and field testing is a totally realistic approach. In addition to these approaches, there is another type of method, theoretical analysis. Such analysis can provide a theoretical guide to the problem, for example, uncompensated gyro biases b_g_ and accelerometer biases b_a_ result in position errors (1/6)b_g_gt^3^ and (1/2)b_a_t^2^ for 2-D navigation, respectively, where t is the time and g is the local gravity value. However, this is an approximate calculation based on a linear motion assumption. Abbott and Powell showed an indication of the achievable performance that can be expected from an in-car GNSS/odometer/gyro system, but it is also based on the assumption of uniform linear motion [12]. There is literature focused on analysis of the system performance through the use of observability analysis [13]. However, these theoretical methods are limited in simple vehicle motion conditions. In practical uses, the complexity of realistic navigation conditions makes the propagation of errors complex in the navigation algorithm, which is difficult to analyze.

This paper is mainly on practical methods; thus, the theoretical analysis approaches are not included. The characteristics of lab calibration, simulation, and field testing are described as followings:

(1) Lab Calibration

The inertial sensor errors can be divided into two types: deterministic (systematic) errors and stochastic errors. Deterministic errors consist of biases, scale factors, inertial axis misalignments, *etc.*, while stochastic errors include noise, bias instabilities, *etc.* Biases and scale factor errors are the dominant deterministic error sources during the INS standalone navigation process. Approximately, for 2D navigation, gyro biases result in position errors proportional to the time cubed. Meanwhile, accelerometer biases and the sensor scale factors introduce position errors proportional to the time squared [14]. Calibration is particularly useful to remove biases and scale factor errors, and provide IMU parameters including bias instabilities, noise densities, non-linearities, temperature sensitivities, *etc.* In order to obtain deterministic and stochastic inertial sensor errors, different calibration methods can be chosen according to application requirements.

Various calibration methods for deterministic errors have been presented. The most widely used type is the approaches based on a turntable [15]. With the references from the turntable, each sensitive axis of every accelerometer can point alternately up and down precisely, and the IMU can be rotated around each gyro axis, both clockwise and counter-clockwise, with accurately-known angles. When a turntable it is not available, scholars have proposed the multi-position method, the basic idea of which can be stated as: the norms of the measured outputs of the accelerometer and gyro clusters are equal to the magnitude of the given specific force (*i.e.*, gravity) and rotation (*i.e.*, the Earth’s rotational rate) inputs [16]. For MEMS IMUs, the main drawback in using the multi-position method is that the Earth’s rotational rate is too weak (15 deg/h) which results in observability problems. For stochastic errors, the commonly used method for determine their models and parameters include autocorrelation analysis, power spectral density (PSD), and the variance techniques (e.g., Allan variance). Refer to [17] for details about stochastic sensor errors and their modeling.

However, lab calibration cannot provide the actual navigation performance precisely [18], as navigation performance can only be deduced by conducting field testing or analyzing the steady state of a Kalman filter and error propagation in the INS mechanization, rather than being evaluated by lab calibration directly [19]. In this situation, only errors that are modeled by the Kalman filter (e.g., biases and scale factors) can be investigated, while the other errors cannot. To evaluate the navigation performance, INS simulation and field testing are two possible approaches.

(2) INS Simulation

INS simulation is one common choice for navigation performance evaluation. It can simulate the ideal navigation information (e.g., reference IMU signals, and reference vehicle moving trajectories), and add sensors errors (obtained from sensor specifications or lab calibration) to the reference signals to generate pseudo-IMU signals. Compared with lab calibration, the simulation method is closer to the evaluation of navigation performance because one can process the simulated signals and evaluate the navigation results. Meanwhile, compared with field tests, simulation has three advantages: (a) simulation is more flexible. Users can design vehicle moving trajectories and IMU data according to requirements [20,21]; (b) through simulation, one can evaluate the impact of one certain factor (e.g., one type of sensor error or vehicle motion condition) [22]; and (c) simulation is cost-effective, as it can be implemented without any hardware cost. However, both vehicle motions and sensor errors are simulated, which may be different from those in actual situation. Even though it is straightforward to simulate the IMU errors, for some types of errors (e.g., temperature variations, long-term drifts, and the stochastic errors), it is difficult to ensure that the simulated values are the same as the corresponding actual sensor errors in practice. Some high-grade MEMS sensors have an embedded temperature drift compensation; however, many low-cost MEMS sensors do not have such a mechanism. In addition, the GNSS signals are artificial, as well.

(3) Field Testing

GNSS/INS field testing is the most realistic and accurate approach to evaluate the performance of IMUs [23,24]. In a field test, IMU errors, GNSS signals, and vehicle motions are all real. However, it takes significant expenses and time to conduct field tests. Furthermore, to cover different trajectories and road conditions, a group of tests is required, which will be even more expensive and time-consuming. Therefore, it is not practical to test every new IMU, especially a low-cost MEMS IMU, through field testing.

To fill the gap among the methods mentioned above, we present a time- and cost-effective way to evaluate the navigation performance of a new IMU. The proposed method is named as “Signal Grafting (SG)”. The SG method is essentially a hybrid simulation and testing method that generates IMU data through signal grafting (*i.e.*, by adding real signals from the tested IMU to signals from a reference IMU). The basic idea behind this implementation is that sensor errors and their stabilities of a higher-grade IMU are several times lower than those of a lower-grade IMU (Table 1 shows the specifications of various grades of IMUs). Therefore, signals from a higher-grade IMU (that has been calibrated in lab to mitigate the majority of deterministic errors) can be regarded as “reference” outputs that do not have sensor errors when compared with signals from a lower-grade IMU.

Compared to the lab calibration method, the SG method is implemented at the navigation performance level, instead of the sensor error level. Compared to the INS simulation method, the SG method uses real signals. Both the real vehicle motions and GNSS signals can be provided during the SG process. Meanwhile, the sensor errors extracted from the IMU data are real, instead of being simulated by using sensor error models. Compared to the field testing method, the SG method is much more efficient and economic.

The main contributions of this research are that it presents a novel SG method, and makes a comprehensive verification on the SG method, including its validity to both medium- and low-grade IMUs. The proposed SG method has several benefits:

(1) It can not only improve the efficiency and flexibility of the experiment, but also save the cost of evaluation. In the SG method, only one set of typical field tests with a higher-grade (e.g., navigation-grade) IMU is needed, and the collected data can be used as reference data to evaluate various IMUs in future. For the IMUs to be tested, only data collected in lab are needed, as such data can be grafted to the reference data to generate the SG IMU data (*i.e.*, IMU signals that are generated by using the SG method and can be used in the same way as those collected in real field tests).
(2) The SG method provides an extra evaluation approach before the implementation of real field tests. This is important because various types of IMUs from different manufacturers come to the market every year. Thus, it is not time- and cost-affordable for a researcher to develop the data-collection hardware platform and algorithm for every IMU, and evaluate them through real tests. However, through the use of the SG method, one can first evaluate an IMU by simply grafting its signals to the reference data, and can decide whether to buy the development kit and test the IMU or not.
(3) It provides a general evaluation process for various IMUs. Specifically, with this method, it is feasible to compare the navigation performances (e.g., the attitude and position results) of different IMUs directly when the signals from these IMUs are grafted to the same set of reference trajectory. In this case, it is similar to the case that different types of IMU were installed on the same point on the same vehicle that moved with the same motion conditions, and in the same navigation environment.
(4) To use the SG method, only one set of real test trajectories (*i.e.*, the reference trajectory) is needed. Thus, one can focus on designing and optimizing this trajectory. Once the reference trajectory has been well-designed (e.g., it covers various types of vehicle motions and experiences different kinds of navigation environments such as open sky, urban canyon, forest, and underground for land-based navigation applications), this trajectory is valuable for the other researchers. The peers can also graft their IMU signals to the reference signal and implement the SG method to evaluate their IMUs.
(5) The SG method can generate datasets under some extreme conditions (e.g., in the condition of extreme temperature or quick temperature variation), which cannot be achieved through real field tests.

Form the point (2) mentioned above, it is notable that the purpose of our research is to provide an extra evaluation approach to guide the choose of a new IMU, instead of totally replacing the field testing method by the proposed method, although the test results in this research indicated that the proposed SG method provided similar results as real field tests.

Additionally, this paper presents and evaluates two strategies for generating the SG data:

(1) Basic-error strategy: grafting the extracted basic sensor errors to the reference data to generate the SG #1 IMU data, and
(2) Full-error strategy: using the real-time function graft the full set of IMU errors (*i.e.*, basic sensor errors + dynamic sensor errors) to the reference data to generate the SG #2 IMU data, and comparing their results.

In this paper, the basic errors are the sensor errors that always exist when the sensor is working, no matter whether the device is moving or not. Examples of the basic errors are biases and noise. On the other hand, the dynamic errors are the sensor errors which show an impact only when the device is moving. Examples of the dynamic errors are scale factor errors and cross-axis sensitivities (*i.e.*, non-orthogonalities).

The outline of this paper is as follows: Section 2 covers a brief description of the SG method. Section 3 describes the experimental setup and data processing results to verify this method. Finally, the summary and conclusions of this study are provided in Section 4.

## 2. Methodology

**Figure 1:**
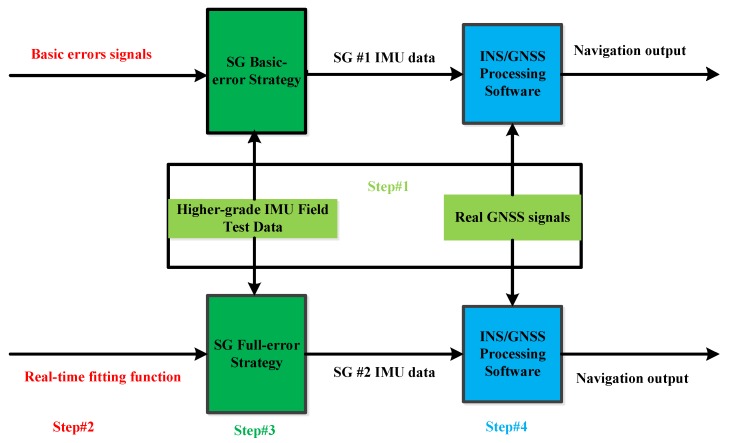
Scheme diagram of the proposed SG method (light green: Step #1; red: Step #2; green: Step #3; light blue: Step #4).

The proposed SG method is a hybrid simulation and testing method that generates IMU data through signal grafting (*i.e.*, adding real signals from the tested IMU to signals from a higher-grade reference IMU). The main steps for the implementation and verification of the method can be described as follows (also shown in Figure 1).

Step #1: collect reference signals (*i.e.*, design the reference trajectory, and conduct field tests with a higher-grade IMU). 

Step #2: obtain in-lab basic errors signals and real-time function from the IMU to be tested.

Step #3: implement signal grafting.

Step #4: process navigation data, and evaluate the solution.

The proposed SG method is effective because Step #1 is the only in-field step, while the other steps are conducted in lab. Furthermore, the SG method is economic because the Step #1 is needed to be implemented only once to obtain the signals from the higher-grade IMU used and the navigation solution provided through the integration of this IMU and GNSS. For the evaluation of every IMU to be tested, one just needs the outcomes from Step #1, instead of implementing Step #1 every time. The four steps are described separately in this section.

### 2.1. Step #1: Reference Signals Generation

It is preferred that the reference trajectory covers various vehicle motion conditions and navigation environments. The higher-grade IMU can provide two types of references: (a) after in-lab calibration, the outputs from the higher-grade IMU can be regarded as “reference” IMU signals that do not have sensor errors when compared to the signals from a lower-grade IMU; moreover, (b) the “reference” navigation solution obtained through the integration of the information from the higher-grade IMU and GNSS. A Kalman filter is used for GNSS/INS integration, and a backward smoothing can be used enhance the integrated navigation solution.

To ensure the reliability of the reference IMU signals and reference trajectory, lab calibration is particularly useful to mitigate the majority of the deterministic errors, including biases, scale factor errors, and non-orthogonalities of the higher-grade IMU [18]. Therefore, in this section, we first introduce the sensor error models and the lab calibration method.

#### 2.1.1. Sensor Error Models

The output of accelerometers and gyros can be written as [14]:

(1) $$\hat{f}=\left[I+S_{a}+N_{a}\right]f+b_{a}+w_{a}$$

(2) $$\hat{\omega }=\left[I+S_{g}+N_{g}\right]\omega +b_{g}+w_{g}$$

where $\hat{f}$ and $\hat{\omega }$ are the error vectors of the accelerometer-derived specific forces and the gyro-derived angular rates, f and ω are the reference specific forces and the reference angular rates, I is the identity matrix, $S_{a}$ and $S_{g}$ are the diagonal matrices containing the scale factor errors, $b_{a}$ and $b_{g}$ are the accelerometer and the gyro biases, $N_{a}$ and $N_{g}$ are the skew-symmetric matrices containing the non-orthogonalities, and $w_{a}$ and $w_{g}$ represent accelerometer and gyro noise.

#### 2.1.2. Lab Calibration

Among many of the calibration methods, the six-position static and rate tests method is most commonly used due to its reliability and simplicity of implementation [14]. This method can be used to get a full set of deterministic sensor errors (*i.e.*, biases, scale factor errors and non-orthogonalities).

To estimate a full set of accelerometer errors, the output of a triad of accelerometers is represented in matrix form by:

(3) [f^xf^yf^z]=[1+SxNyxNzxNxy1+SyNzyNxzNyz1+Sz             baxbaybaz]︸M[fxfyfz1]

where the diagonal S elements are the scale factors, the off diagonal m elements represent the non-orthogonalities and the b components are the biases, $f_{x}$, $f_{y}$, and $f_{z}$ are the x-, y-, and z- axis components of f in formula (1), respectively, and ${\hat{f}}_{x}$, ${\hat{f}}_{y}$, and ${\hat{f}}_{z}$ are the components of $\hat{f}$. In the calibration scheme, successively, the axis of each accelerometer is kept pointing upwards and downwards for a period of time and the ideal acceleration can be represented as follows:

(4) $$f_{1}^{'}=\left[\begin{array}{c} g\\ 0\\ 0 \end{array}\right]\;f_{2}^{'}=\left[\begin{array}{c} -g\\ 0\\ 0 \end{array}\right]\;f_{3}^{'}=\left[\begin{array}{c} 0\\ g\\ 0 \end{array}\right]\;f_{4}^{'}=\left[\begin{array}{c} 0\\ -g\\ 0 \end{array}\right]\;f_{5}^{'}=\left[\begin{array}{c} 0\\ 0\\ g \end{array}\right]\;f_{6}^{'}=\left[\begin{array}{c} 0\\ 0\\ -g \end{array}\right]\;$$

Then, the design matrix can be denoted by A and the measured acceleration of the accelerometer is denoted by U.

(5) $$A=\left[\begin{array}{c} f_{1}^{'}\\ 1 \end{array}\;\begin{array}{c} f_{2}^{'}\\ 1 \end{array}\;\begin{array}{c} f_{3}^{'}\\ 1 \end{array}\;\begin{array}{c} f_{4}^{'}\\ 1 \end{array}\;\begin{array}{c} f_{5}^{'}\\ 1 \end{array}\;\begin{array}{c} f_{6}^{'}\\ 1 \end{array}\right]$$

(6) $$U=\left[\begin{array}{cccc} \begin{array}{ccc} u_{1} & u_{2} & u_{3} \end{array} & u_{4} & u_{5} & u_{6} \end{array}\right]$$

In this case, the column vector of the U matrix should be:

(7) $$u_{1}={\left[\begin{array}{c} {\hat{f}}_{x}\\ {\hat{f}}_{y}\\ {\hat{f}}_{z} \end{array}\right]}_{X-\text{upwards}}\;u_{2}={\left[\begin{array}{c} {\hat{f}}_{x}\\ {\hat{f}}_{y}\\ {\hat{f}}_{z} \end{array}\right]}_{X-\text{downwards}}\;$$

$u_{3}$, $u_{4}$, $u_{5}$, and $u_{6}$ are similar with $u_{1}$ and $u_{2}$. Then, the M matrix can be estimated by the least-square method:

(8) $$M=U\cdot A^{T}{\left(A\cdot A^{T}\right)}^{-1}$$

Different from the calculation of the accelerometer errors, it is better to estimate the gyro errors through a two-step method, instead of using the least-square method directly. The first step is to calculate the biases using static data. The other step is to calculate the scale factor errors and non-orthogonalities with dynamic data.

The bias of the i-axis (i = x, y, z) gyro can be calculated by:

(9) $$b_{gi}=\frac{l_{i-\text{upwards}}+l_{i-\text{downwards}}}{2}$$

where $l_{i-\text{upwards}}$ and $l_{i-\text{downwards}}$ are the gyro outputs when the axis points upwards and downwards, respectively.

The scale factors of the i-axis gyro can be estimated using the same idea as the six-position method:

(10) $$S_{gi}=\frac{L_{i-\text{clockwise}}-L_{i-\text{antilockwise}}}{2L_{\mathrm{ref}}}-1$$

where $S_{gi}$ is gyro scale factor of the i-axis gyro, $L_{i-\text{clockwise}}$ and $L_{i-\text{antilockwise}}$ represent the angle derived by the integration of the i-axis gyro output when the IMU is rotated around this axis by $L_{\mathrm{ref}}$ clockwise and counter-clockwise, respectively. 

The non-orthogonalities between i-axis and j-axis can be estimated by the output of the j-axis when the IMU is rotated around i-axis in both clockwise and counter-clockwise direction:

(11) $$n_{ij}=\frac{L_{j-\text{clockwise}}-L_{j-\text{antilockwise}}}{2L_{\mathrm{ref}}}$$

where $n_{ij}$ are the non-orthogonalities of i-axis to j-axis, $L_{j-\text{clockwise}}$ and $L_{j-\text{antilockwise}}$ are the output of the j-axis when the IMU is rotated around the i-axis by $L_{\mathrm{ref}}$ in the clockwise or counter-clockwise direction.

### 2.2. Step #2: Obtain IMU Basic Error Signals and Real-Time Fitting Functions in-Lab

Two types of IMU data. *i.e.*, the static data and the dynamic data, can be collected in this step. The static data of the tested IMU is used to extract the basic error signals of the IMU, while the dynamic data of the tested IMU is collected to get the real-time fitting function of the IMU.

(a) Basic errors signals

In this step, we collect IMU outputs under static conditions for a time period. During static periods, the effect of noises can be reduced through averaging the sensor outputs. For static data, the effect of noise can be reduced through averaging the sensor outputs. The level of sensor errors caused by noise [25], can be calculated by:

(12) $$D_{\text{accuracy}\_b}=\frac{\sigma_{RW}}{\sqrt{t_{\text{static}}}}$$

where $\sigma_{RW}$ is the angular random walk (ARW) coefficient for gyros or velocity random walk (VRW) coefficient for accelerometers, $D_{\text{accuracy}\_b}$ is the level of gyro or accelerometer errors, and $t_{\text{static}}$ is the static time.

**Figure 2:**
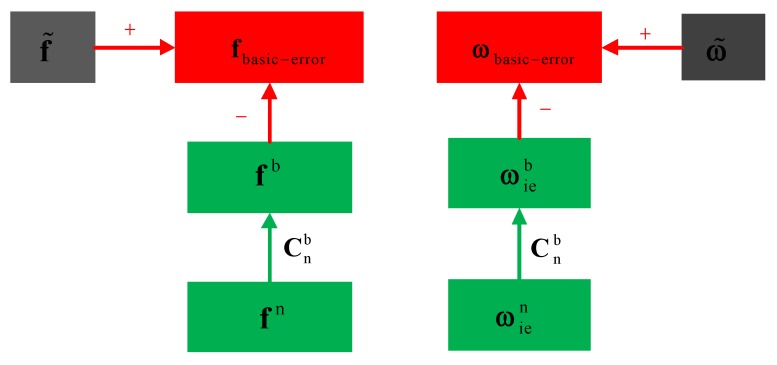
Block diagram of static error signals generation.

To extract the basic error signals of the tested IMU, the influences of the gravity and the Earth rate should be removed from the static IMU data by:

(13) $$f_{\text{basic}-\text{error}}=\tilde{f}-f^{b}$$

(14) $$\omega_{\text{basic}-\text{error}}=\tilde{\omega }-\omega_{ie}^{b}$$

where $f_{\text{basic}-\text{error}}$ and $\omega_{\text{basic}-\text{error}}$ are the basic error signals of accelerometers and gyros. The basic errors are comprised of sensor biases, noises, and other sensor errors exist when the IMU is static. As shown in Table 1, biases are one main component in the basic errors. $\tilde{f}$ and $\tilde{\omega }$ are the actual outputs of accelerometers and gyros, and $f^{b}$ and $\omega_{ie}^{b}$ are the theoretical outputs of accelerometers and gyros. The generation of the basic error signals can be described as follows (also shown in Figure 2).

To transform the IMU outputs from the body frame (*i.e.*, b-frame) to the navigation frame (*i.e.*, n-frame), the direction cosine matrix (DCM) is calculated by:

(15) $$C_{b}^{n}=\left(\begin{array}{ccc} \cos\theta \cos\psi & -\cos\phi \sin\psi +\sin\phi \sin\theta \cos\psi & \sin\phi \sin\psi +\cos\phi \sin\theta \cos\psi \\ \cos\theta \sin\psi & \cos\phi \cos\psi +\sin\phi \sin\theta \sin\psi & -\sin\phi \cos\psi +\cos\phi \sin\theta \sin\psi \\ -\sin\theta & \sin\phi \cos\theta & \cos\phi \cos\theta \end{array}\right)$$

(16) $$C_{n}^{b}={\left(C_{b}^{n}\right)}^{T}$$

where $C_{b}^{n}$ is the DCM from the b-frame to the n-frame. The sign ${\left(\right)}^{T}$ represents the transposition of a matrix. ϕ, θ, and ψ are the roll, pitch, and heading angles, respectively.

(17) $$f^{b}=C_{n}^{b}f^{n}$$

(18) $$f^{n}=\left(\begin{array}{l} 0\\ 0\\ g \end{array}\right)$$

$f^{b}$ is the specific force in the b-frame (*i.e.*, the theoretical output of accelerometers), $f^{n}$ is the specific force in the n-frame, and g is the normal gravity on the local position.

(19) $$\omega_{ie}^{b}=C_{n}^{b}\omega_{ie}^{n}$$

(20) $$\omega_{ie}^{n}=\left(\begin{array}{l} \omega_{e}\cos\varphi \\ \begin{array}{cc} & 0 \end{array}\\ -\omega_{e}\sin\varphi \end{array}\right)$$

$\omega_{ie}^{b}$ is the angular rate of the Earth frame (*i.e.*, e-frame) relative to the inertial frame (*i.e.*, i-frame) in the b-frame, $\omega_{ie}^{n}$ is the angular rate of the e-frame relative to the i-frame in the n-frame, $\omega_{e}$ is the angular rate of the Earth, and φ is the latitude.

(b) Real-time fitting function

In this step, we collect the accelerometer outputs under motion conditions that have different accelerations, and collect the gyro outputs under motion conditions that have various angular rates. The motion conditions with various accelerations or angular rates can be provided through the use of a turntable. To provide such motions, a common way is to control the turntable to make the IMU experience various attitudes to ensure the accelerometer axes sense different specific forces, and control the turntable to bring in rotations with various angular rates around each gyro axis. Additionally, the time length of the specific dynamic condition should be long enough to ensure that the influence of sensor biases is more significant than that of the noise.

Once the dynamic data is obtained, a fitting function is used to add the full set of error signals from the tested IMU to higher-end reference IMU signals. The fitting function is obtained with the aid of MATLAB^®^ Curve Fitting Toolbox^TM^ from the MathWorks ^TM^ Inc. Equation (21) is a function command using the Curve Fitting Toolbox:

(21) p=polyfit(x,y,n)

where x is the theoretical output vector of accelerometers or gyros, y is the actual output vector of accelerometers or gyros. n and p are the highest power and coefficient vector of the polynomial, respectively.

### 2.3. Step #3: Signal Grafting (SG)

**Figure 3:**
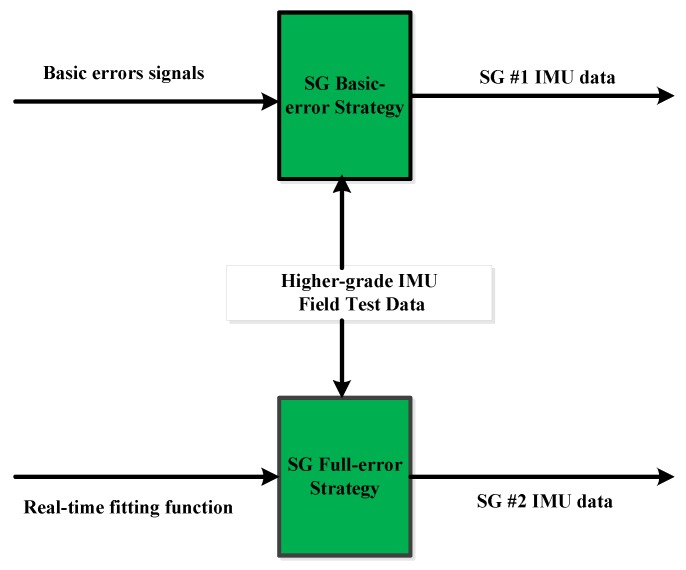
Scheme diagram of the SG implementation.

This step grafts the extracted sensor errors from Step #2 to the reference data by following the strategies described at the end of the introduction, specifically, the basic-error strategy which grafts the basic errors extracted from static IMU data, and the full-error strategy which uses the full set of errors that includes both the basic errors and the dynamic errors generated from the fitting function. The corresponding generated IMU data are denoted as SG #1 and SG #2 IMU data, respectively. The implementation of SG is shown in Figure 3.

The performance of two strategies were compared to show the impact of both the basic and dynamic errors on the SG method. In principle, the basic-error strategy can only reflect the impact of basic IMU errors, which may have major impact on the performance of navigation applications that have low or medium vehicle dynamics. For high dynamic applications, dynamic IMU errors may become dominant and the full-error strategy may become appropriate. Dynamics refer to the scenarios with correlation motion variations; here, taking the speed for an example, in the low dynamic, the vehicle speed range is 0 to 20 km/h and in the medium dynamic, the vehicle speed range is 20 to 40 km/h.

### 2.4. Step #4: Data Processing and Performance Evaluation

This step processes the SG IMU datasets generated by the SG method, and evaluates the navigation performance by comparing the result with the reference trajectory obtained by using the integration of a higher-grade IMU and GNSS. Meanwhile, in this research, we design another performance evaluation approach by collecting the real field data of the tested IMU together with the higher-grade IMU data in Step #1, processing the real tested IMU data with the same navigation algorithm, and comparing the result with that obtained by using the SG IMU data.

This section describes the data processing algorithm, and gives a structure of the loosely-coupled method for fusing GNSS and INS information. In such integration, the GNSS-derived position information is updating the MEMS sensors through a Kalman filter while the IMU is used to provide the navigation information during GNSS signal outages. Refer to [26] for details about the loosely coupled GNSS/INS integration Kalman filter algorithm.

(a) Kalman Filter Dynamic Models

The INS error models with respect to the computer frame (*i.e.*, c-frame, locally level frame at the computed position) are used as the Kalman filter dynamic models. The used error model is called the ψ-angle error model since the attitude errors are expressed in terms of the ψ-angle [27,28].

(22) $$\begin{array}{l} \delta {\dot{r}}^{c}=-\omega_{ec}^{c}\times \delta r^{c}+\delta v^{c}\\ \delta {\dot{v}}^{c}=\delta g^{c}-(2\omega_{ie}^{c}+\omega_{ec}^{c})\times \delta v^{c}+f^{c}\times \psi +C_{b}^{p}\delta f^{b}\\ \dot{\psi }=-(\omega_{ie}^{c}+\omega_{ec}^{c})\times \psi -C_{b}^{p}\delta \omega_{ib}^{b} \end{array}$$

where, $\delta {\dot{r}}^{c}$, $\delta {\dot{v}}^{c}$ and $\dot{\psi }$ are the time derivative of position errors, velocity errors and attitude errors, respectively; $\delta g^{c}$ is the gravity error projected to the c-frame; $\omega_{ie}^{c}$ is the angular rate of the e-frame relative to the i-frame, projected to the c-frame; $\omega_{ec}^{c}$ is the angular rate of the c-frame relative to the e-frame, projected to the c-frame; $\delta \omega_{ib}^{b}$ and $\delta f^{b}$ are the gyro and accelerometer output errors; and $C_{b}^{p}$ is the DCM from the b-frame to the platform frame (*i.e.*, p-frame).

(b) Kalman Filter Measurement Models

GNSS position and/or velocity can be used as the Kalman filter measurement updates in the data processing algorithm. The concrete measurements vectors of the Kalman filter are the difference between the INS-derived position and/or velocity and the GNSS information, *i.e.*,

(23) $$z=\left(\begin{array}{c} r_{\text{INS}}-r_{\text{GNSS}}\\ v_{\text{INS}}-v_{\text{GNSS}} \end{array}\right)$$

**Figure 4:**
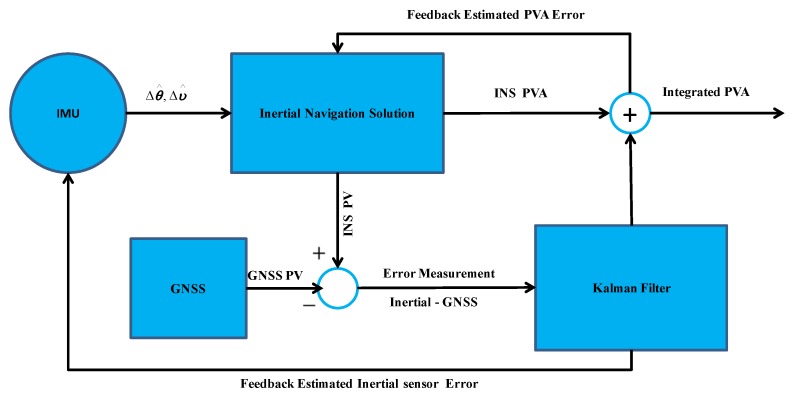
Block diagram of GNSS/INS Integration Kalman filter; PVA represents position, velocity, and attitude.

The information fusion is realized through the loosely-coupled and closed-loop implementation of GNSS/INS integration, as shown in Figure 4. Here, the estimated IMU errors are fed back to correct the INS. Refer to [26] for details about correlation of INS errors.

Essentially, the proposed SG method can be regarded as a balance between the INS simulation and field testing methods. The SG method has unique advantages over the three conventional IMU evaluation methods mentioned in Section 1:

■ Compared to the lab calibration method, the SG method can implement the evaluation at the navigation performance level, instead of the sensor error level.
■ Compared to the INS simulation method, the SG method uses real signals. First, the vehicle motion types and parameters, navigation scenarios, and GNSS signals are all real. Meanwhile, sensor errors are extracted from the real data of the tested IMU, instead of being simulated by using sensor error models.
■ Compared to the field testing method, the SG method is much more efficient and economic. In the SG method, only one set of typical field tests with a higher-grade IMU is needed and can be used to evaluate various IMUs in future. Only data collected in the lab are needed from the tested IMUs; this implementation saves the hardware cost and time for the both the standalone INS and hardware integration with GNSS. Moreover, the SG method can provide datasets collected under some extreme conditions (e.g., under the condition of extreme temperature or quick temperature variation), which cannot be achieved through real field tests.

## 3. Experimental Verification

### 3.1. Test Nomenclature

Before introducing the tests, it is necessary to explain the terms used, including:

(a) IMU_SG#1: IMU data generated by using the basic-error strategy that considers basic IMU errors.
(b) IMU_SG#2: IMU data generated by using the full-error strategy that considers the full set of IMU errors.
(c) IMU_REAL: real outputs of the tested IMU.
(d) IMU_REF: real outputs of the higher-grade IMU.
(e) SOL_1: generating navigation solutions through the integration of the IMU_SG#1 and real GNSS data.
(f) SOL_2: generating navigation solutions through the integration of the IMU_SG#2 and real GNSS data.
(g) SOL_REAL: generating navigation solutions through the integration of the real outputs of the tested IMU and real GNSS data.
(h) SOL_REF: generating navigation solutions through the integration of the real outputs of the higher-grade IMU and real GNSS data.
(i) ERR _1: absolute differences between SOL_1 and SOL_REF solutions.
(j) ERR_2: absolute differences between SOL_2 and SOL_REF solutions.
(k) ERR_REAL: absolute differences between SOL_REAL and SOL_REF solutions.
(l) DIFF _1: result of ERR_1 divided by ERR_REAL.
(m) DIFF _2: result of ERR_2 divided by ERR_REAL. DIFF _2 is an **external** indicator, which is used to reflect the performance of the proposed SG method by comparing its results with those from field testing. The difference between DIFF_2 and DIFF_1 indicate the **internal** differences between results when using the same SG method but following two SG strategies:
  (24) $$DIFF\_1\;=\;\left|SOL\_1-SOL\_REAL\right|\;*\frac{1}{ERR\_REAL}$$
  (25) $$DIFF\_2=\left|SOL\_2-SOL\_REAL\right|*\frac{1}{ERR\_REAL}$$

### 3.2. Test Description

SOL_REAL is not needed for the purpose of implementing the SG method; it is simply used as a reference for evaluating whether the SG method can provide similar results as real field testing. SOL_REF is utilized to provide a reference navigation trajectory.

To evaluate the results, we first obtained the navigation errors of SOL_1, SOL_2 and SOL_REAL by comparing their navigation results with that of SOL_REF. Then, we compared the SOL_1 and SOL_2 errors with the SOL_REAL errors to illustrate whether the SG results match the real one. Meanwhile, we made a comparison between the SOL_1 and SOL_2 results to show whether it is necessary to conduct the full-error strategy, because the implementation of the full-error strategy is more time-consuming, and requires a turntable.

**Figure 5:**
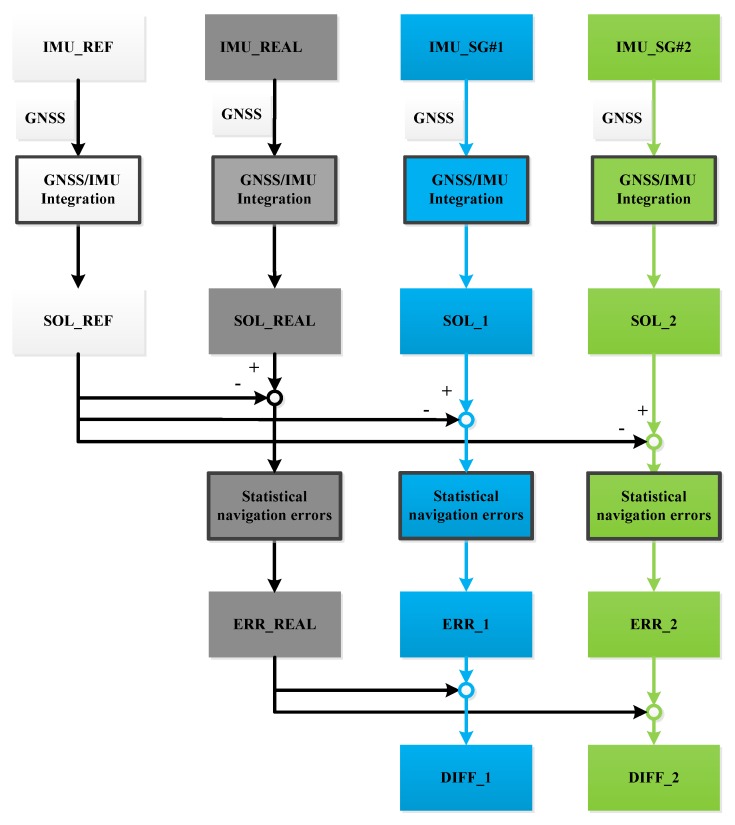
Scheme diagram of data processing and result evaluation.

**Figure 6:**
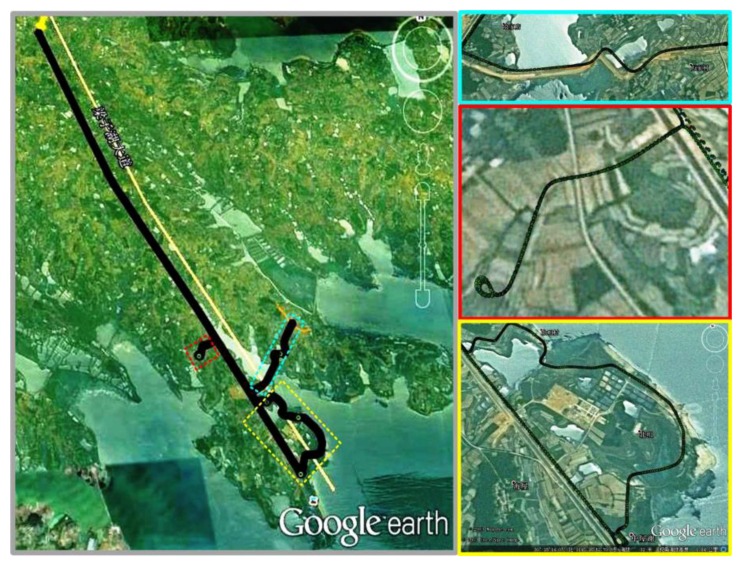
Description of trajectory.

Data processing and result evaluation of the SG can be described as follows (also shown in Figure 5):

To verify the validity of the SG method, three land-based field tests that lasted for 60~75 min were carried out in an open-sky environment with optimum GNSS signals. In Figure 6, three subfigures on the right illustrate Trajectories #1, #2, and #3, respectively, while the sub-figure on the left shows the area of these tests in the same map. 

In each test, the vehicle was equipped with a GNSS receiver, a navigation-grade IMU, and two different IMUs provided by Whhan MAP Space Time Navigation Technology Inc. [29]. The navigation-grade IMU was a MP-POS830 with a gyro bias of under 0.01 deg/h. Two IMUs to be tested included a tactical-grade IMU (MP-POS310) and a MEMS IMU (MP-POS1100). Table 1 shows the specifications of each IMU.

The GNSS/INS data processing software, Cinertial 1.0, developed by the Navigation Group of the GNSS Research Center at Wuhan University [30], was used to process the raw IMU data with carrier-phase differential GNSS (DGNSS) solutions in a loosely-coupled architecture. The position, velocity and attitude estimation results were obtained by integrating the IMU_SG#1, IMU_SG#2 and IMU_REAL data with DGNSS. Meanwhile, the post-processed integration result of the POS830 IMU and DGNSS was used to provide the reference position, velocity, and attitude, so as to calculate the errors of SOL_1, SOL_2, and SOL_REAL. Furthermore, in order to compare the essential performance of each IMU by itself, a set of GNSS outages of 60 s were artificially added in the data processing process [31]. The position drifts during these GNSS outage periods were checked.

### 3.3. Results and Analysis

For each tested IMU, the SG results were compared with the corresponding real field test results by plotting the position, velocity, and attitude errors in one representative sample test. Furthermore, the statistical results of their differences were summarized in the following tables [32,33].

#### 3.3.1. Tactical-grade IMU: MP-POS310

The parameter setting of the system noise matrix (Q) and measurement noise matrix (R) for the integration of POS310 and DGNSS are shown in Equations (26) and (27), respectively.

(26) $$Q=\left[\begin{array}{ccccccc} 0_{3\times 3} & 0_{3\times 3} & 0_{3\times 3} & 0_{3\times 3} & 0_{3\times 3} & 0_{3\times 3} & 0_{3\times 3}\\ 0_{3\times 3} & \text{diag}\left\{{\left(ARW\right)}^{2}\right\} & 0_{3\times 3} & 0_{3\times 3} & 0_{3\times 3} & 0_{3\times 3} & 0_{3\times 3}\\ 0_{3\times 3} & 0_{3\times 3} & \text{diag}\left\{{\left(VRW\right)}^{2}\right\} & 0_{3\times 3} & 0_{3\times 3} & 0_{3\times 3} & 0_{3\times 3}\\ 0_{3\times 3} & 0_{3\times 3} & 0_{3\times 3} & \text{diag}\left\{\frac{2\sigma_{gb}{}^{2}}{T_{gb}}\right\} & 0_{3\times 3} & 0_{3\times 3} & 0_{3\times 3}\\ 0_{3\times 3} & 0_{3\times 3} & 0_{3\times 3} & 0_{3\times 3} & \text{diag}\left\{\frac{2\sigma_{ab}{}^{2}}{T_{ab}}\right\} & 0_{3\times 3} & 0_{3\times 3}\\ 0_{3\times 3} & 0_{3\times 3} & 0_{3\times 3} & 0_{3\times 3} & 0_{3\times 3} & \text{diag}\left\{\frac{2\sigma_{gs}{}^{2}}{T_{gs}}\right\} & 0_{3\times 3}\\ 0_{3\times 3} & 0_{3\times 3} & 0_{3\times 3} & 0_{3\times 3} & 0_{3\times 3} & 0_{3\times 3} & \text{diag}\left\{\frac{2\sigma_{as}{}^{2}}{T_{as}}\right\} \end{array}\right]$$

(27) $$R=\left[\begin{array}{cc} {\left(GNSS\_P\text{std}\right)}_{3\times 3}^{2} & 0_{3\times 3}\\ 0_{3\times 3} & {\left(GNSS\_V\text{std}\right)}_{3\times 3}^{2} \end{array}\right]$$

where ARW are the angle random walk of gyros, VRW are the velocity random walk of accelerometers, $\sigma_{gb}$ and $\sigma_{ab}$ are the gyro and accelerometer bias instabilities, $\sigma_{gs}$ and $\sigma_{as}$ are the gyro and accelerometer scale factor errors, $T_{gb}$, $T_{ab}$, $T_{gs}$, and $T_{as}$ are the correlation time of the random processes, GNSS_Pstd and GNSS_Vstd are the GNSS position and velocity measurement errors, and $0_{3\times 3}$ represents a 3×3 zero matrix.

The elements in the matrix Q (*i.e.*, initial values of sensor output uncertainties) are set according to the corresponding specifications of the exploited sensors. The elements in the R matrix should be determined according to the actual measurement precision of GNSS.

**Figure 7:**
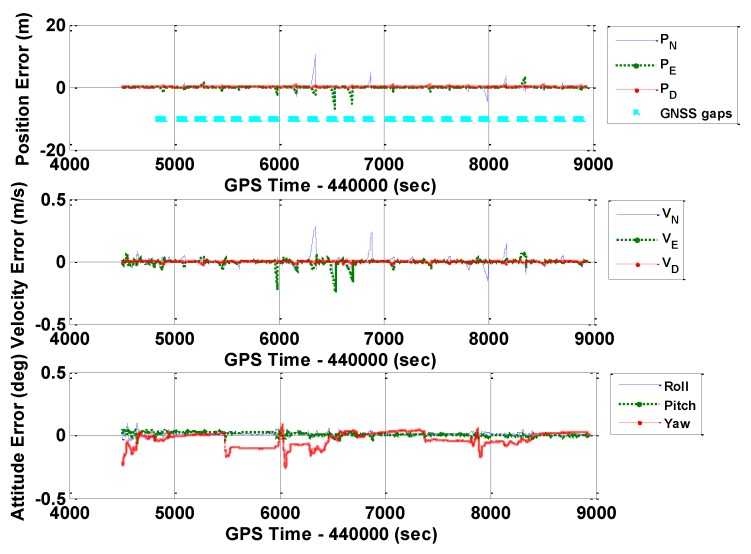
Navigation errors of GNSS/INS with frequent GNSS outages (POS310, ERR_1).

**Figure 8:**
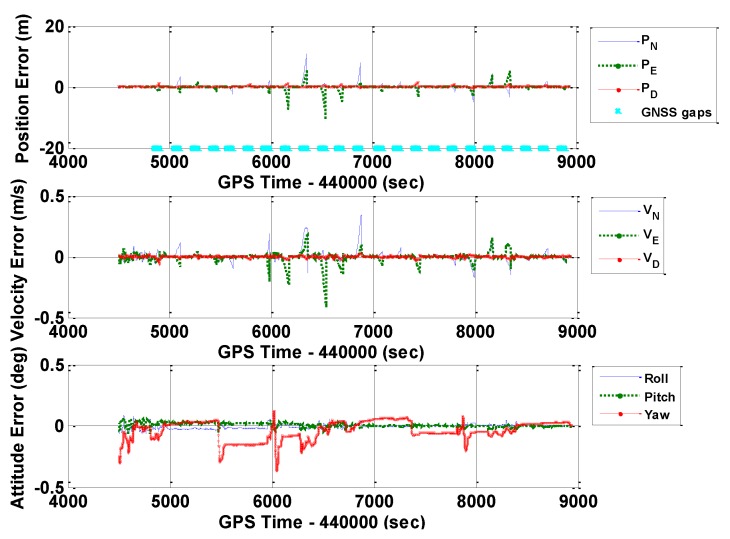
Navigation errors of GNSS/INS with frequent GNSS outages (POS310, ERR_2).

**Figure 9:**
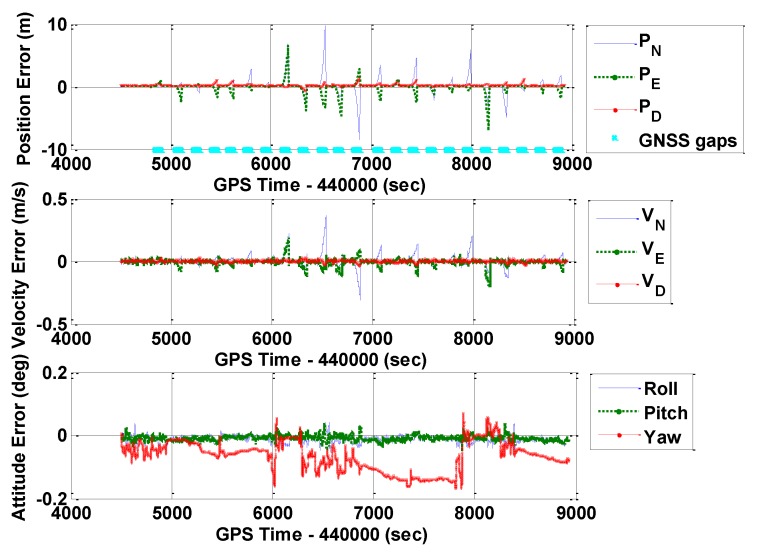
Navigation errors of GNSS/INS with frequent GNSS outages (POS310, ERR_REAL).

As mentioned in Section 3.1, GNSS outages (60 s) were added in the data processing to evaluate the performance of a standalone IMU [31]. Figure 7, Figure 8 and Figure 9 show the POS310 position, velocity, and attitude errors when integrated with GNSS, but had GNSS outages by using ERR_1, ERR_2, and ERR_REAL, respectively. The cyan dots indicate the periods of GNSS outages. The significant drifts during the outages reflected the performance of the standalone POS310 IMU when using the IMU_SG#1 and IMU_SG#2 signals and real outputs. Specifically, for results of ERR_1, ERR_2, and ERR_REAL, the maximum values were all approximately 10 m for position errors and 0.4 m/s for velocity errors. The magnitudes and features of the ERR_1 and ERR_2 navigation errors generally matched with that of ERR_REAL. These outcomes indicated the validity of the proposed SG method for the evaluation of a tactical-grade IMU.

Table 2 illustrates the following outcomes (when using the tactical-grade IMU POS310 during GNSS outage periods lasted for 60 s):

■ When navigating with a standalone, tactical-grade IMU for 60 s, the navigation accuracy was 5 m for horizontal positions, 0.7 m for vertical position, 0.03 deg for horizontal attitudes, and 0.1 deg for the heading. The navigation accuracy was described by the root mean square (RMS) values of the navigation errors (*i.e.*, differences between navigation results and the corresponding results from the reference system). The RMS values are calculated by using the INS navigation errors during multiple GNSS outage periods. Among 23 GNSS outage periods, the maximum values of position drifts were 10.350 m, 6.827 m, and 0.897 m along the north, east, and down directions, respectively. The maximum attitude drifts reached 0.053 deg, 0.043 deg, and 0.165 deg for roll, pitch, and heading, respectively.
■ The difference between the ERR_2 and ERR_REAL solutions was below 5% for both position and attitude errors. This outcome illustrated that when navigating this tactical-grade IMU by itself over a periods of 60 s, the proposed SG method can achieve the similar performance to field tests that utilized real IMU data.
■ The difference between the ERR_1 and ERR_2 solutions were under 7% for horizontal attitude errors, and nearly 20% for position errors and 28% for the heading error. This phenomenon indicated that the differences between the basic-error strategy (*i.e.*, considering only basic IMU errors) and the full-error strategy (*i.e.*, considering the full set of IMU errors) results were significant for both position and heading in this case. When comparing results with and without GNSS, it was found that the dynamic sensor errors had larger impact on the SG method when there was no GNSS updates.

#### 3.3.2. MEMS IMU: MP-POS1100

Compared with POS310, the POS1100 data were processed with the same navigation algorithm but with different parameter settings. The parameter setting of the system noise matrix (Q) according to the corresponding specifications of the MP-POS1100. 

**Figure 10:**
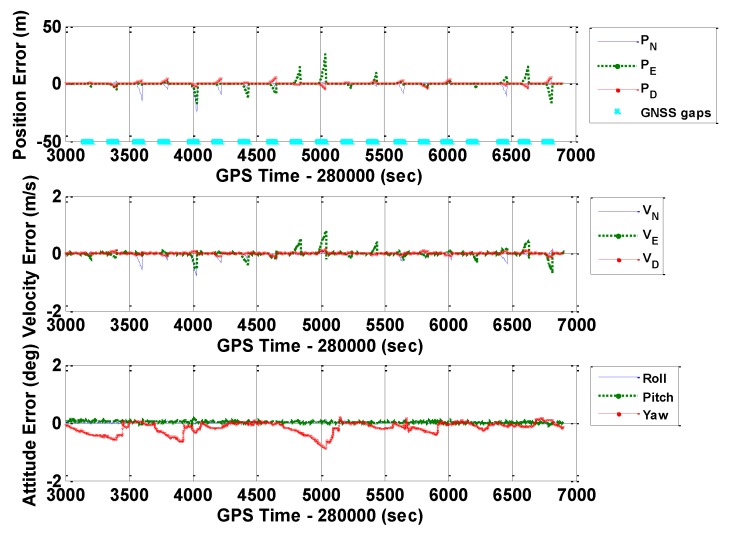
Navigation errors of GNSS/INS with frequent GNSS outages (POS1100, ERR_1).

**Figure 11:**
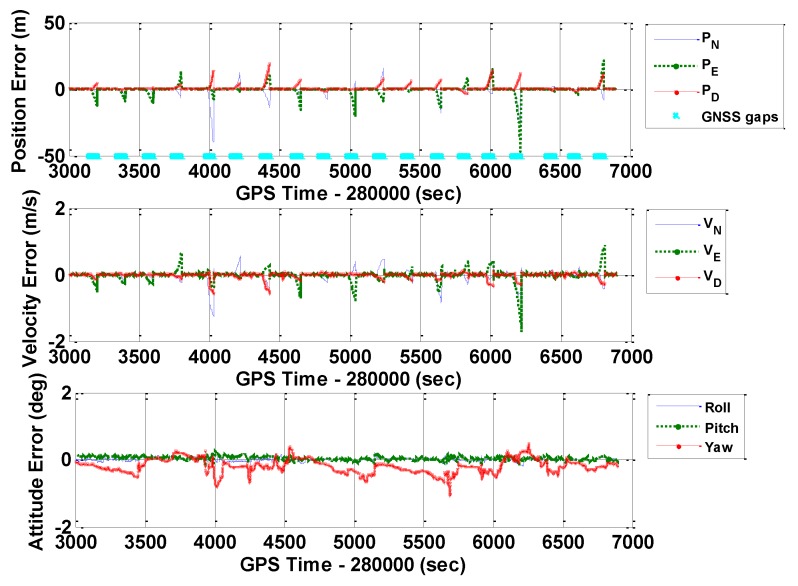
Navigation errors of GNSS/INS with frequent GNSS outages (POS1100, ERR_2).

**Figure 12:**
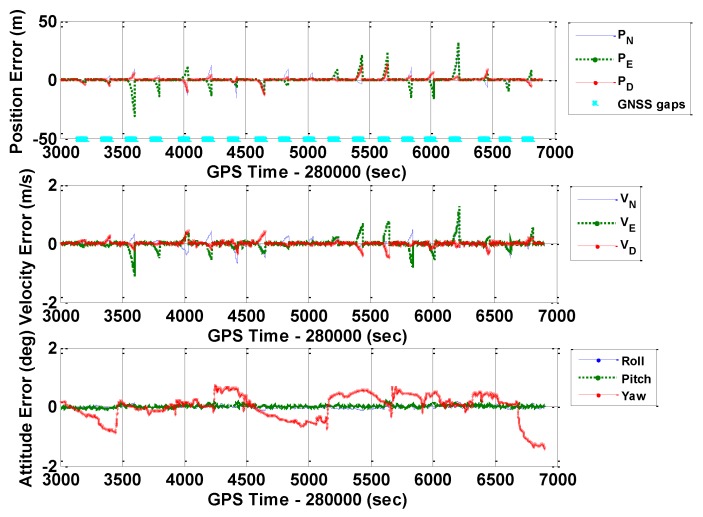
Navigation errors of GNSS/INS with frequent GNSS outages (POS1100, ERR_REAL).

Figure 10, Figure 11 and Figure 12 illustrate the navigation drifts during GNSS outage periods, respectively. Additionally, Table 3 shows the statistical results.

Table 3 illustrates the following outcomes (when using the MEMS IMU POS1100 during GNSS outage periods lasted for 60 s):

■ When navigating with this MEMS IMU for 60 s, the navigation accuracy (RMS) increased to 20 m for horizontal positions, 8 m for vertical position, 0.12 deg for horizontal attitudes, and 0.5 deg for the heading. Such values were much larger than the corresponding values in Table 2 (with a tactical-grade IMU). These outcomes make sense when comparing the sensor errors of these two IMUs. Among all GNSS outage periods, the maximum values of position drifts were 23.930 m, 25.858 m, and 5.072 m along the north, east, and down directions, respectively. The maximum attitude drifts reached 0.152 deg, 0.142 deg, and 0.902 deg for roll, pitch, and heading, respectively.
■ The differences between the ERR_2 and ERR_REAL solutions were below 20% for position and horizontal attitude errors, and 30% for heading errors. These differences were larger than those in Table 2, but not significant in general. When analyzing the trend of the heading errors, we found that the differences mainly occurred during the periods when the vehicle was moving straight with a constant velocity. Thus, the reason for the occurrence of differences may be explained as follows: GNSS/INS integrated navigation systems suffer from poor observability of the heading angle when the vehicle moved with weak dynamics [13,33,34]. Such an observability issue may cause large uncertainty of the heading estimation result.
■ The largest difference between the ERR_1 and ERR_2 occurred in the vertical position errors (33%). The difference in DIFF_1 and DIFF_2 results further supported the outcome that the dynamic sensor errors may have significant impact on the SG method when there was no GNSS update.

Comparing Table 2 and Table 3, one obtains the following outcomes:

■ For the tested tactical-grade IMU, the difference between the SG and field testing errors were below 4.2% for all position and attitude errors when there were frequent GNSS outages (lasted for 60 s). For the tested MEMS IMU, the maximum differences between the SG and field testing errors was 19.4% for positions, 19.4% for horizontal attitudes, and 26.7% for heading, when there were frequent GNSS outages (lasted for 60 s). The reason for the occurrence of differences is the differences between the grafted signals and real signals. For the tested tactical-grade IMU, the sensor error has good stability, the grafted signals can basically reflect real changes in the degree and level. However, for the tested MEMS IMU, the stability of the sensor error is poorer; thus, the result is susceptible to interference and change. (Table 1 shows the specifications of various grades of IMUs).

According to the above results and analyses, the differences between the results of the SG method and field testing were within an acceptable range for a rough evaluation of the performance of both tactical-grade and low-cost MEMS IMUs. The performance of the SG method is even promising when considering the fact that the SG method was implemented without any field tests by using the IMU to be tested, and when considering that part of these differences might be caused by some uncontrollable factors when implementing the field tests. Thus, the proposed SG method can be promoted for IMU performance evaluation because it can reflect the final navigation performance, be operated in a convenient and efficient way, and reduce the expense and time of the field tests. Meanwhile, the differences between ERR_1 and ERR_2 results indicated that it is necessary to take dynamic errors into account to exploit the performance of the SG method.

## 4. Conclusions

This paper presents a time- and cost-effective IMU evaluation method (*i.e.*, the SG method) and makes comprehensive verifications and analyses on its performance. Three road tests involving two grades of IMUs were conducted to verify the feasibility of the SG method. Furthermore, the SG results were compared with real field testing results with frequent GNSS outages. For the tested tactical-grade IMU, the difference between SG and field testing statistical errors were below 4.2% for all position and attitude errors when there were frequent GNSS outages (lasting for 60 s). For the tested MEMS IMU, the maximum differences between SG and field testing errors was 19.4% for positions, 19.4% for horizontal attitudes, and 26.7% for heading when there were frequent GNSS outages. Therefore, the differences between the results of the SG method and field testing were in an acceptable range for a rough evaluation of the performance of both tactical-grade and low-cost MEMS IMUs. The performance of the SG method is even promising when considering the fact that the SG method was implemented without any field tests by using the IMU to be tested.

Therefore, this research provides an efficient and extremely low-cost approach to predict the performance of the IMU in the lab before the implementation of real field tests. Furthermore, the SG method is a general evaluation approach, which is feasible to compare the navigation performances of different IMUs directly by grafting the signals from these IMUs to the same set of reference trajectories. Thus, the well-designed reference data (which covers various types of vehicle motions and experiences different kinds of navigation environments) from one researcher can be valuable for the peers, and be useful to compare the solutions from different research groups.

This paper also compared the performance of two SG strategies and indicated that it is necessary to take dynamic errors into account to exploit the performance of the SG method. Future works will focus on improving the SG method by grafting more sensor errors, such as non-orthogonalities and non-linearities, and evaluating the method with airborne and marine navigation data.

## Figures and Tables

**Table 1:** Specifications of the tested IMUs.

Sensor	Characteristic	IMU		
		MP-POS830	MP-POS310	MP-POS1100
	Grade	Navigation	Tactical	MEMS
**Gyro**	**Bias instability (deg/h)**	0.005	0.5	<10
	**White noise (ARW, deg/sqrt (h))**	0.0022	0.05	0.15
	**Scale Factor** **(ppm)**	10	300	1000
**Accel.**	**Bias instability (mg)**	0.025	0.5	<1
	**White noise (VRW, m/s/sqrt (h))**	0.00075	0.1	0.06
	**Scale Factor** **(ppm)**	10	300	1000

**Table 2:** Statistical navigation errors of INS (POS310).

	Position Errors (RMS, m)		Attitude Errors (RMS, deg)		
	Horizontal *	Vertical	Roll	Pitch	Heading
ERR_1	3.758	0.518	0.027	0.022	0.066
ERR_2	4.857	0.651	0.029	0.025	0.093
ERR_REAL	4.688	0.651	0.029	0.024	0.097
DIFF_1	19.84%	20.43%	6.90%	8.33%	31.96%
**DIFF_2**	**3.60%**	**0.00%**	**0.00%**	**4.17%**	**4.12%**

* “Horizontal” = $\sqrt{P_{N}^{2}+P_{E}^{2}}$, “Vertical” = $P_{D}$.

**Table 3:** Statistical navigation errors of INS (POS1100).

	Position Errors (RMS, m)		Attitude Errors (RMS, deg)		
	Horizontal *	Vertical	Roll	Pitch	Heading
ERR_1	12.983	3.131	0.077	0.063	0.334
ERR_2	20.422	7.842	0.113	0.117	0.378
ERR_REAL	17.657	6.566	0.100	0.098	0.516
DIFF_1	26.47%	52.31%	23.00%	35.71%	35.27%
**DIFF_2**	**15.66%**	**19.43%**	**13.00%**	**19.39%**	**26.74%**

* “Horizontal” = $\sqrt{P_{N}^{2}+P_{E}^{2}}$, “Vertical”=$P_{D}$.
